# Supplementary material for: Endophytes from African Rice (Oryza glaberrima L.) Efficiently Colonize Asian Rice (Oryza sativa L.) Stimulating the Activity of Its Antioxidant Enzymes and Increasing the Content of Nitrogen, Carbon, and Chlorophyll
Source: Microorganisms. 2021 Aug 11;9(8):1714. doi: 10.3390/microorganisms9081714 (PMC8398951; doi:10.3390/microorganisms9081714)
Supplement: Supplementary file 1 [file microorganisms-09-01714-s001.zip › Supplementary_Materials_Bianco_30.07.2021/TableS1.pdf]

Table S1. Antibiotic resistance of N-fixing endophytes isolated from *Oryza glaberrima* plants

**Antibiotic**[a]	***Citrobacter sp. BDA59-3***	***Kasakonia pseudosacchari BDA62-3***	***Enterobacter sacchari BDA86-11***	***Klebsiella pasteuri BDA134-6***	***Kosakonia oryzendophytica BDA137-1***	***Enterobacter sp. BDAM41-2***
**Ampicillin (60)**	+++	+++	+++	+++	++	+++
**Carbenicillin (25)**	+++	+++	n.d.	+++	+++	+++
**Erythromycin (15)**	++	++	+	+++	+++	+++
**Fosfomycin (25)**	++	+++	+++	+++	+++	+
**Fosfomycin (50)**	++	+++	+++	+++	+++	+
**Kanamycin (10)**	+++	+++	-	n.d.	-	++
**Nalidixic acid (5)**	-	n.d.	-	-	-	-
**Nalidixic acid (50)**	-	n.d.	-	-	-	-
**Novobiocin (50)**	+	+++	+	+	+	+++
**Neomycin (15)**	-	-	-	-	+++	-
**Penicillin G (25)**	++	+++	+++	n.d.	+++	+++
**Rifampicin (50)**	-	-	n.d.	-	n.d.	-
**Spectinomycin (25)**	-	-	-	-	+	-
**Spectinomycin (200)**	-	-	-	-	-	-
**Streptomycin (50)**	-	-	-	-	-	-
**Streptomycon (200)**	-	-	-	-	-	-
**Tetracycline (5)**	-	+	-	-	-	+
**Tetracycline (10)**	-	-	.	-	-	-
**Vancomycin (20)**	+++	+++	+++	+++	+++	+++
**Vancomycin (40)**	++	++	+++	+++	+++	++

[a]The numbers in brackets represent the concentration (in $\mu g\ ml^{-1}$) of antibiotic used.

+, weakly resistant; ++, moderately resistant; +++, strongly resistant; -, not resistant; n.d., not determined
