# Supplementary material for: Endophytes from African Rice (Oryza glaberrima L.) Efficiently Colonize Asian Rice (Oryza sativa L.) Stimulating the Activity of Its Antioxidant Enzymes and Increasing the Content of Nitrogen, Carbon, and Chlorophyll
Source: Microorganisms. 2021 Aug 11;9(8):1714. doi: 10.3390/microorganisms9081714 (PMC8398951; doi:10.3390/microorganisms9081714)
Supplement: Supplementary file 1 [file microorganisms-09-01714-s001.zip › Supplementary_Materials_Bianco_30.07.2021/TableS2.pdf]

Table S2. Identification and characterization of culturable endophytes isolated from *Oryza glaberrima* plants

Isolate	Accession number[a]	Closest relative (accession number)[b]	Order	Phylum	Similarity (%)	IAA production[a]	*nifH*[b] gene	*acds*[b] gene
**BDA11-1**	MT241164	*Aeromonas hydrophila strain F_5* (MG428760.1)	Aeromonadales	Proteobacteria	99.8	-	-	-
**BDA11-2**	MT241165	*Stenotrophomonas maltophilia strain IAE99* (MK415049.1)	Xanthomonadales	Proteobacteria	99.4	-	-	-
**BDA11-3**	MT241166	*Stenotrophomonas maltophilia strain BPS2* (MK786692.1)	Xanthomonadales	Proteobacteria	100	-	-	-
**BDA11-4**	MT241167	*Pseudomonas mosselii strain AB1816* (MK045617.1)	Pseudomonadales	Proteobacteria	99.4	-	-	-
**BDA59-1**	MT241168	*Pseudomonas moraviensis strain JAS18* (KF528828.1)	Pseudomonadales	Proteobacteria	100	-	-	-
**BDA59-2**	MT241169	*Stenotrophomonas maltophilia strain B641* (MK301171.1)	Xanthomonadales	Proteobacteria	100	-	-	-
**BDA59-3**	CP063427.1	*Phytobacter diazotrophicus strain UAEU22* (CP051548.1)	Enterobacterales	Proteobacteria	98.9	+	+	-
**BDA59-4**	MT241171	*Klebsiella pneumoniae strain AR_0080* (LC386023.1)	Enterobacterales	Proteobacteria	98.7	+	-	-
**BDA62-2**	MT241172	*Klebsiella pneumoniae strain TBMAX84* (MK834722.1)	Enterobacterales	Proteobacteria	99.5	+	-	-
**BDA62-3**	CP063425.1	*Kosaconia pseudosacchari strain TL13* (MN607214.1)	Enterobacterales	Proteobacteria	100	+	+	-
**BDA62-4**	MT241174	*Pantoea stewartii strain EP200* (MG778874.1)	Enterobacterales	Proteobacteria	99.2	+	-	-
**BDA73-4**	MT241175	*Exiguobacterium indicum strain E19* (MH150815.1)	Bacillales	Firmicutes	99.8	-	-	-
**BDA73-5**	MT241176	*Bacillus pumilus isolate KMP123-MS1* (LT978407.1)	Bacillales	Firmicutes	100	-	-	-
**BDA73-7**	MT241177	*Pseudomonas nitroreducens strain L4* (MH196902.1)	Pseudomonadales	Proteobacteria	99.9	-	-	-
**BDA73-8**	MT241178	*Paenibacillus silvae strain CH03* (MK618628.1)	Bacillales	Firmicutes	99.9	+	-	-
**BDA73-9**	MT241179	*Arthrobacter woluwensis strain ICMP 20856* (MG786378.1)	Actinomycetales	Actinobacteria	100	-	-	-
**BDA73-10**	MT241180	*Acinetobacter oryzae strain WJB33* (KU877635.1)	Pseudomonadales	Proteobacteria	100	+	-	-
**BDA73-12**	MT241181	*Exiguobacterium indicum strain DSAM 62* (MH819520.1)	Bacillales	Firmicutes	100	-	-	-
**BDA86-1**	MT241182	*Paenibacillus hunanensis strain KH3* (LC025995.1)	Bacillales	Firmicutes	96.7	+	-	-
**BDA86-2**	MT241183	*Pseudomonas otitidis strain T8* (MG283318.1)	Pseudomonadales	Proteobacteria	99.5	+	-	-
**BDA86-3**	MT241184	*Klebsiella oxytoca strain R1* (MK801235.1)	Enterobacterales	Proteobacteria	99.7	+	-	-
**BDA86-4**	MT241185	*Pantoea agglomerans strain KEK 45* (KY486226.1)	Enterobacterales	Proteobacteria	99.6	+	-	-
**BDA86-5**	MT241186	*Pseudomonas monteilii strain HE_P46* (MK235233.1)	Pseudomonadales	Proteobacteria	99.8	-	-	-
**BDA86-6**	MT241187	*Enterobacter roggenkampii strain SQUCC_LB11* (MK583581.1)	Enterobacterales	Proteobacteria	100	+	-	+
**BDA86-7**	MT241188	*Bacillus cereus strain N12* (MF099842.1)	Actinomycetales	Firmicutes	99.5	-	-	-
**BDA86-10**	MT241189	*Microbacterium hydrothermale strain mICRO84* (MK696251.1)	Actinomycetales	Actinobacteria	100	+	-	-
**BDA86-11**	MT241190	*Enterobacter sacchari strain NN208E* (HQ204315.1)	Enterobacterales	Proteobacteria	100	+	+	-

Table SX *Continued*

**Isolate**	**Accession number**[a]	**Closest relative (accession number)**[b]	**Order**	**Phylum**	**Similarity (%)**	**IAA production**[a]	***nifH***[b] **gene**	***acds***[b] **gene**
**BDA89-1**	MT241191	*Enterobacter ludwigii strain HBT4* (MG571688.1)	Enterobacterales	Proteobacteria	100	+	-	-
**BDA89-2**	MT241192	*Sphingobacterium multivorum strain BZN8* (MK886726.1)	Sphingobacteriales	Bacteroidetes	100	-	-	-
**BDA89-3**	MT241193	*Enterobacter asburiae strain 162* (MH910266.1)	Enterobacterales	Proteobacteria	99.8	+	-	-
**BDA107-2**	MT241194	*Enterobacter asburiae strain N15165* (MK389324.1)	Enterobacterales	Proteobacteria	99.7	+	-	-
**BDA107-3**	MT241195	*Pantoea* sp. *A1128* (JX266309.1)	Enterobacterales	Proteobacteria	99.9	+	-	-
**BDA107-5**	MT241196	*Acinetobacter seifertii strain 34M* (MK874918.1)	Pseudomonadales	Proteobacteria	100	-	-	-
**BDA107-6**	MT241197	*Enterobacter roggenkampii strain HBUAS53378* (MK818764.1)	Enterobacterales	Proteobacteria	99.6	+	-	-
**BDA107-7**	MT241198	*Chryseobacterium gleum strain CIFRI-SRM4* (MK770613.1)	Flavobacteriales	Bacteroidetes	99.6	+	-	-
**BDA107-9**	MT241199	*Enterobacter asburiae strain U4* (KC434995.1)	Enterobacterales	Proteobacteria	99.9	+	-	-
**BDA107-11**	MT241200	*Enterobacter ludwigii strain AO_03* (KX760131.1)	Enterobacterales	Proteobacteria	99.9	+	-	-
**BDA107-12**	MT241201	*Enterobacter* sp. *UIWRF0555* (KR189731.1)	Enterobacterales	Proteobacteria	99.9	+	-	-
**BDA107-13**	MT241202	*Enterobacter* sp. *3-1t* (EU543690.1)	Enterobacterales	Proteobacteria	99.5	+	-	-
**BDA134-1**	MT241203	*Enterobacter* sp. *strain MK17* (KP974272.1)	Enterobacterales	Proteobacteria	100	+	-	-
**BDA134-2**	MT241204	*Stenotrophomonas maltophilia strain IAE127* (MK414820.1)	Xanthomonadales	Proteobacteria	100	-	-	-
**BDA134-4**	MT241205	*Enterobacter asburiae strain BKA4* (MK530091.1)	Enterobacterales	Proteobacteria	100	+	-	-
**BDA134-5**	MT241206	*Pseudomonas putida strain AEGB5* (MK829424.1)	Pseudomonadales	Proteobacteria	100	-	-	-
**BDA134-6**	CP064784.1	*Klebsiella pasteurii strain SPARK1489C1 (MN104667.1)*	Enterobacterales	Proteobacteria	100	+	+	-
**BDA134-8**	MT241208	*Aeromonas hydrophila strain CP2V8-03* (MK534033.1)	Aeromonadales	Proteobacteria	99.9	+	-	-
**BDA137-1**	MT241209	*Kosakonia oryzendophytica strain Tm.Vt-SE.Av01* (MK039407.1)	Enterobacterales	Proteobacteria	99.8	+	+	-
**BDA137-3**	MT241210	*Pseudomonas taiwanensis strain Pt-CW19* (MK880383.1)	Pseudomonadales	Proteobacteria	99.6	+	-	-
**BDA137-4**	MT241211	*Pseudescherichia vulneris JCM 2130* (LC382121.1)	Enterobacterales	Proteobacteria	99.7	-	-	-
**BDA137-5**	MT241212	*Enterobacter cloacae strain PYLG* (KY767543.1)	Enterobacterales	Proteobacteria	99.9	+	-	-
**BDA137-6**	MT241213	*Pantoea stewartii strain JZ82* (KY194282.1)	Enterobacterales	Proteobacteria	99.7	+	-	-
**BDA137-7**	MT241214	*Pantoea agglomerans strain TKW_28* (KY486214.1)	Enterobacterales	Proteobacteria	99.6	+	-	-
**BDA137-8**	MT241215	*Enterobacter* sp. *DL4.7* (JQ912516.1)	Enterobacterales	Proteobacteria	99.9	+	-	-
**BDA137-9**	MT241216	*Ralstonia mannitolilytica strain PMB-2* (MH890459.1)	Burkholderiales	Proteobacteria	100	-	-	+
**BDA137-10**	MT241217	*Stenotrophomonas maltophilia strain IAE123* (MK414816.1)	Xanthomonadales	Proteobacteria	99.6	-	-	-
**BDA137-11**	MT241218	*Klebsiella pneumoniae strain TBMAX84* (MK834722.1)	Enterobacterales	Proteobacteria	99.9	+	-	-

Table SX *Continued*

Isolate	Accession number[a]	Closest relative (accession number)[b]	Order	Phylum	Similarity (%)	IAA production[a]	*nifH*[b] gene	*acds*[b] gene
**BDA137-12**	MT241219	*Enterobacter* sp. *MK17* (KP974272.1)	Enterobacterales	Proteobacteria	99.8	+	-	-
**BDA137-13**	MT241220	*Microbacterium laevaniformans strain RT83* (MK014262.1)	Actinomycetales	Actinobacteria	99.8	+	-	+
**BDA137-15**	MT241221	*Pantoea agglomerans strain TKW_51* (KY486229.1)	Enterobacterales	Proteobacteria	100	+	-	
**BDA137-16**	MT241222	*Microbacterium* sp. *B05* (HM209349.1)	Actinomycetales	Actinobacteria	99.6	+	-	+
**BDA138-1a**	MT241223	*Pantoea agglomerans strain D51_MA4R* (MK883157.1)	Enterobacterales	Proteobacteria	100	+	-	-
**BDA138-1b**	MT241224	*Acinetobacter baumannii strain LIM-PHCN-016* (MK840990.1)	Actinomycetales	Proteobacteria	99.8	-	-	-
**BDA141-1**	MT241225	*Leclercia adecarboxylata strain NIBSM_OsR11* (KY930711.1)	Enterobacterales	Proteobacteria	99.5	+	-	-
**BDA141-2**	MT241226	*Chryseobacterium endophyticum strain CC-YTH209* (NR_156142.1)	Flavobacteriales	Bacteroidetes	99.9	+	-	-
**BDAM41-2**	MT241227	*Enterobacter sp. strain ERR 833* (MF442271.1)	Enterobacterales	Proteobacteria	100	+	+	+
**BDAM41-6**	MT241228	*Stenotrophomonas rhizophila strain ELM4B45* (KP860574.1)	Xanthomonadales	Proteobacteria	99.2	+	-	+
**BDAM42-1**	MT241229	*Enterobacter* sp. *CZGRN4* (KJ184910.1)	Enterobacterales	Proteobacteria	99.8	-	-	-
**BDAM42-2**	MT241230	*Stenotrophomonas pavanii strain WZN-1* (KY000522.1)	Xanthomonadales	Proteobacteria	99.8	-	-	-
**BDAM42-3**	MT241231	*Klebsiella pneumoniae strain SISX20* (MK780048.1)	Enterobacterales	Proteobacteria	100	+	-	-
**BDAM42-4**	MT241232	*Leclercia adecarboxylata strain UQCH 006* (MK214731.1)	Enterobacterales	Proteobacteria	99.8	+	-	-

[a]GenBank accession number for the nucleotide sequence deposited in the National Center for Biotechnology Information database.

[b]To identify the isolated endophytes, the 16S rRNA gene fragments were PCR-amplified by using universal primers, inserted into a commercial cloning vector, and the positive clones sequenced. The obtained sequences were compared with those deposited in GenBank database (www.ncbi.nlm.nih.gov/BLAST/) using the BLASTN algorithm (BLAST Local Alignment Search Tolls).

[c]Bacterial cultures were grown over-night in LB medium containing L-tryptophan. The IAA produced and released in the culture supernatant was quantified by using the Salkowski reagent.

[d]The *nifH* and *acdS* genes fragments PCR-amplified by using the DNA of *Herbaspirillum seropedicae* z67 and *Sinorhizobium meliloti* 1021 were used as positive controls for the identification of nitrogen-fixing and ACC-deaminase-producing endophytes.
