# Supplementary material for: Endophytes from African Rice (Oryza glaberrima L.) Efficiently Colonize Asian Rice (Oryza sativa L.) Stimulating the Activity of Its Antioxidant Enzymes and Increasing the Content of Nitrogen, Carbon, and Chlorophyll
Source: Microorganisms. 2021 Aug 11;9(8):1714. doi: 10.3390/microorganisms9081714 (PMC8398951; doi:10.3390/microorganisms9081714)
Supplement: Supplementary file 1 [file microorganisms-09-01714-s001.zip › Supplementary_Materials_Bianco_30.07.2021/TableS3.pdf]

Table S3. Genome size and genomic features of N-fixing endophytes isolated from *Oryza glaberrima* plants

**Genome metrics**	***Kasakonia pseudosacchari* BDA62-3**	***Klebsiella pasteurii* BDA134-6**	***Citrobacter* sp. BDA59-3**
**Mean Coverage**	141X	299X	191X
**Genome size (bp)**	5,003,047 bp	6,061,544 bp	5,349,552 bp
**Number of replicons**	2	2	1
**Length of replicons**	chr: 4,909,996bp pBDA62-3: 93,051 bp	chr: 5,912,274 bp; pBDA134-6: 149,270 bp	chr: 5,349,552 bp
**GC%**	53.98	55.44	53.25
**Number of genes**	4790	5612	5073
**Number of CDS**	4683	5499	4962
**CDS assigned to UniProtKB**	2694	4084	3537
**rRNA**	22	25	22
**CRISPR arrays (repeat regions)**	0	1	3
**tRNA**	84	87	85
**tmRNA**	1	1	1
**Methylated DNA motifs (m6A; *, m4C)**	GATC; CTCGAG; HGATCGAGNNNNNNNB	GATC; VSAGCTSS*	GATC; CCTCAG; CTGAAG; CCCANNNNNNNTTG; CAANNNNNNNTGGG
