# Supplementary figures and images for: Endophytes from African Rice (Oryza glaberrima L.) Efficiently Colonize Asian Rice (Oryza sativa L.) Stimulating the Activity of Its Antioxidant Enzymes and Increasing the Content of Nitrogen, Carbon, and Chlorophyll
Source: Microorganisms. 2021 Aug 11;9(8):1714. doi: 10.3390/microorganisms9081714 (PMC8398951; doi:10.3390/microorganisms9081714)

*Kosakonia pseudosacchari* sp. BDA62-3

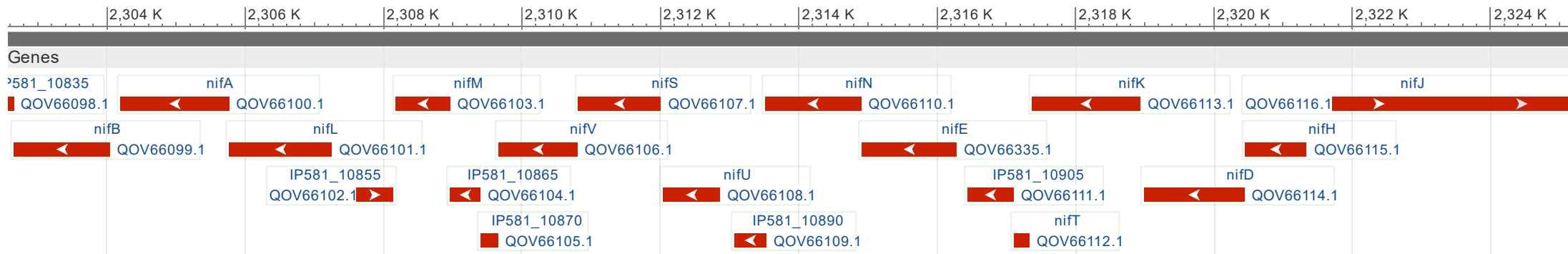

*Klebsiella pasteurii* BDA134-6

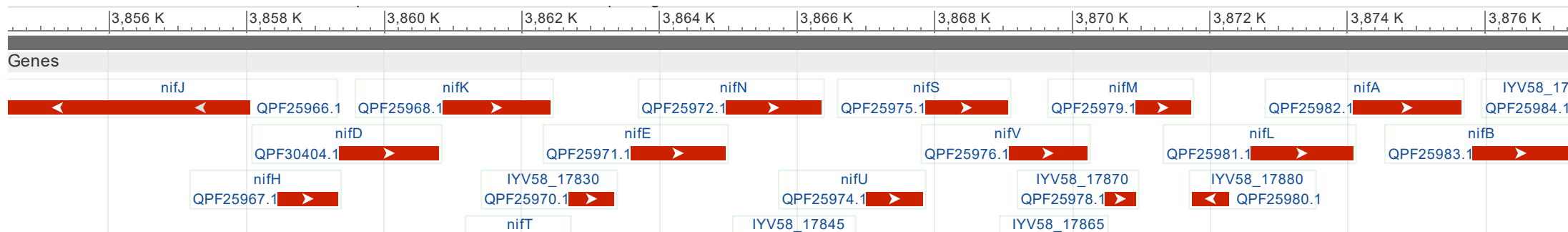

*Citrobacter* sp. BDA59-3

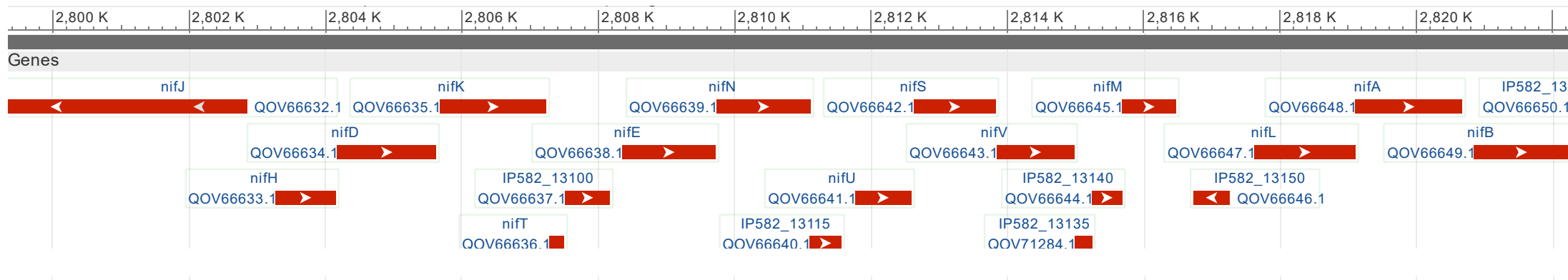

Supplement: Supplementary file 1 [file microorganisms-09-01714-s001.zip › Supplementary_Materials_Bianco_30.07.2021/Figure S1.pdf]
